# Improvement of Ultrasonic Pulse Generator for Automatic Pipeline Inspection

**DOI:** 10.3390/s18092950

**Published:** 2018-09-05

**Authors:** Noé Amir Rodríguez-Olivares, José Vicente Cruz-Cruz, Alejandro Gómez-Hernández, Rodrigo Hernández-Alvarado, Luciano Nava-Balanzar, Tomás Salgado-Jiménez, Jorge Alberto Soto-Cajiga

**Affiliations:** 1 Center for Engineering and Industrial Development (CIDESI), Santiago de Queretaro, Queretaro 76125, Mexico; jcruz@posgrado.cidesi.edu.mx (J.V.C.-C.); algomez@posgrado.cidesi.edu.mx (A.G.-H.); rodrigo.hernandez@cidesi.edu.mx (R.H.-A.); lnava@cidesi.edu.mx (L.N.-B); tsalgado@cidesi.edu.mx (T.S.-J.); 2 Academic Group of Automation and Control, Technological University of the State of Queretaro (UTEQ), Santiago de Queretaro, Queretaro 76148, Mexico

**Keywords:** ultrasonic pulse generator, pipeline inspection gauge, PID, neural network, autoregressive exogenous model, simultaneous optimization of several responses

## Abstract

This paper presents the improvement of an ultrasonic pulse generator for a pipeline inspection gauge (PIG), which uses 64 transducers for inspecting distances up to 100 km with an axial resolution fixed at 3 mm and variable speeds between 0 and 2 m/s. An ultrasonic pulse generator is composed of a high-voltage (HV) MOSFETs, driver logic and an HV power supply. We used a DC-HV DC converter device as the HV power supply because it reduces the size of the ultrasound system considerably. However, pipeline geometry and inspection effects such as hammer and shock cause a variable pulse repetition frequency (PRF), producing voltage drops, poor quality of the HV pulse generated, failures in the dimensioning of defects and damage to devices by over-voltage. Our improvement is to implement a control scheme to maintain the high quality of the HV regardless of the variable PRF. To achieve this, we characterized three transfer functions of the DC-HV DC converter, varying the connected load to 10%, 45% and 80%. For the characterization, we used the least squares technique, considering an autoregressive exogenous (ARX) model. Later, we compared three control schemes: (1) proportional-integral-derivative (PID) tuned by simultaneous optimization of several responses (SOSR), (2) PID tuned by a neural network (NN) and (3) PI tuned by the analytical design method (ADM). The metrics used to compare the control schemes were the recovery time, the maximum over-voltage and the excess energy when the shock and hammer effects happen to occur. Finally, to verify the improvement of the HV pulser, we compared the ultrasonic pulses generated for various frequencies and amplitudes using the pulse generator with and without the control scheme.

## 1. Introduction

Every specific application of ultrasonic non-destructive testing (NDT) determines the parameters used to design the ultrasonic pulse generator [1,2]. In the case of automatic carbon-steel pipeline inspection, the pipeline inspection gauge (PIG) uses piezoelectric transducers due to their simplicity, sensitivity and cost [3]; but the electrical impedance of these transducers hinders the high-voltage (HV) pulse generator circuit design [4]. The transducers work in a pulse-echo mode, and their excitation voltage is set between −100 V and −20 V to ensure that ultrasound echoes are received with sufficient energy [5]. To comply with international standards [6,7], the PIG must inspect the complete pipeline circumference every 3 mm, and the pulse repetition frequency (PRF) of one transducer must not be higher than 600 Hz. One ultrasonic pulse generator excites several transducers through a multiplexer device; therefore, its PRF is higher than 600 Hz. The PRF is variable and non-controllable because it is related to the PIG speed, which varies for the pipeline geometry and inspection effects such as the hammer and shock. The HV power supply is one of the devices most affected by the mentioned speed changes since it transmits energy to all transducers, and a small change in its output voltage causes a series of problems such as: (a) variable amplification of the signal received, which increases existing noise; (b) damage to the components of the pulse generator circuit by over-voltage; (c) failures in wall thickness estimation [8], because the echoes between the inner and the outer wall of the pipe are related to some internal defects in the material, and the amplitude of these echoes allows the determination of the size of the defect found relative to the beam field [9,10]; and (d) null-control of the delivered power.

PIGs check carbon-steel pipelines used for the distribution of oil and water [11]. If the pipeline is in the order of kilometers in length, the PIG carries out an automatic inspection, where the circumferential inspection is made based on external markers and measurements from odometers and gyroscopes that allow the determination of the displacement of the PIG [12]. The angle beam and straight beam are the ultrasonic nondestructive evaluation (NDE) technologies most commonly used in automatic inspection of pipelines [13]. Many PIGs use the ultrasonic pulse-echo method (straight beam technology) to perform the automatic inspection of the pipeline because it gives better results than manual inspection or other techniques used to determine the safety of the pipeline [14]. The pulse-echo method allows volumetric inspection, which is possible because the mechanical waves are able to travel through the fluid and the walls of the pipeline. These waves allow technicians to detect internal and superficial defects, such as cracks that occur parallel to the surface, as well as corrosion and defects within the body of the pipeline [15]. The echo flight-time from both the inner and outer pipeline walls indicates the wall thickness.

**Figure 1:**
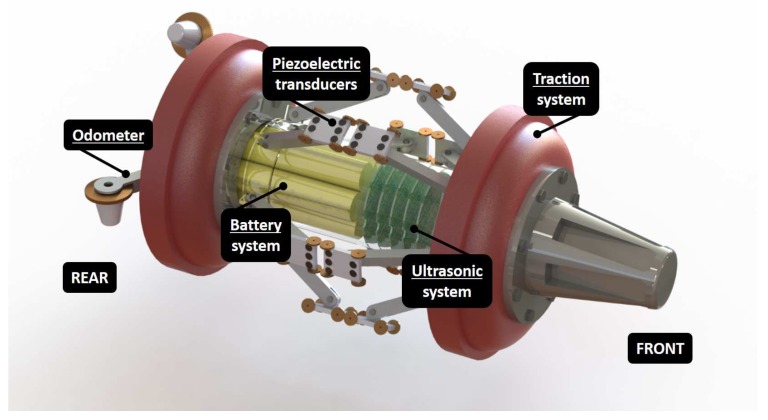
Pipeline inspection gauge (PIG) in development for automatic pipeline inspection.

The PIG must be able to inspect distances up to 100 km at speeds of 0–2 m/s [6,7,16]. The speed of the PIG inside the pipeline varies due to the geometry of the pipeline, the pressure of the fluid, girth welds and factors such as the movements of the Earth and other external effects. These factors disfigure the pipeline diameter and cause the PIG to get stuck for short intervals, producing the hammer and shock effects. In the case of the girth welds, there is one between every two adjacent standard pipe segments, so the hammer and shock effects cannot be ignored [17,18,19]. The hammer effect occurs when the pipeline delivers high inspection speeds [20]. Figure 1 shows our PIG in development; its traction system works through cups, which help it to be propelled by the fluid inside the pipeline. The ultrasonic system maps the whole circumference of the pipeline using 64 piezoelectric transducers (PT) of 5 MHz.

**Figure 2:**
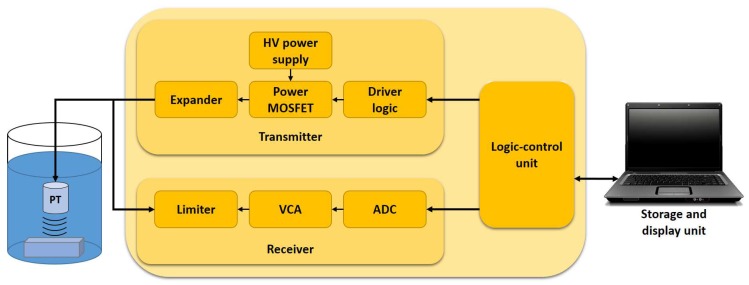
Typical architecture of an ultrasound system for immersion inspection.

Figure 2 shows a typical ultrasound system for NDT, which is composed of an ultrasonic pulse generator or transmitter, a receiver, transducers, a logic-control unit, a storage system and a display unit [2,21,22]. The ultrasonic pulse generator consists of an expander, power MOSFETs, driver logic and an HV power supply. The receiver is composed of a limiter, a variable control amplifier (VCA), and an analog to digital converter (ADC). In our case, one ultrasound system has already been validated to measure thicknesses [23], and it has been used to develop a specialized technique for the on-line reduction of signals [24]. However, performing tests on the laboratory piping circuits, we found that the ultrasonic system has serious problems maintaining the HV excitation at a fixed level due to the changes in speed. The HV control in the ultrasonic pulse generator is necessary considering that our final goal is to use the ultrasonic system for automatic pipeline inspection.

**Figure 3:**
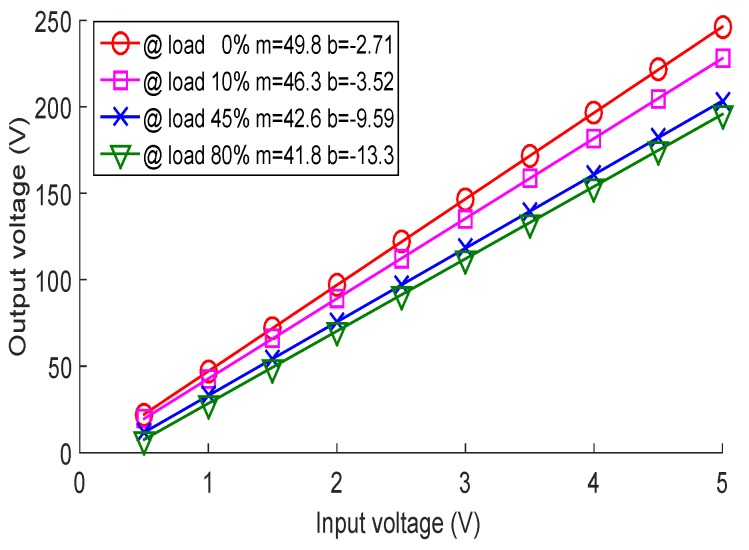
Variation of the output voltage of the HV DC converter device in relation to the input voltage and the connected load. VCA, variable control amplifier.

We used the DC-HV DC converter device EMCO Q02 (XP Power, Singapore) [25] like the HV power supply with the purpose of reducing the size of the ultrasonic pulse generator. The main advantage of the DC-HV DC converter is the small form factor, which makes it useful not only for our small ultrasonic pulser, but also renders it ideal for use in light sources, printers, capacitor charging and other applications, so the control of this device could be useful in other key research areas. Although some DC-HV DC converters are already commercialized with an internal closed-loop control, the quality of the output voltage can be improved by employing an external control scheme that not only regulates the output voltage, but also works as an element for monitoring. Having a control system that monitors the reflected power is very important when estimating the delivered power in ultrasound systems [26]. Figure 3 shows the behavior of the DC-HV DC converter device EMCO Q02 with the input voltage and the percentage of the connected load. For the characterization of the behavior of the DC-HV DC converter device, we used ten values of fixed voltage, starting at 0.5 V and increasing in units of 0.5 V until reaching an input of 5 V. At each input voltage value, we connected at the output precision resistors that represented 10%, 45% and 80% of the static connected load. We interpreted the load variation as the change in the PRF or the number of connected piezoelectric transducers.

The DC-HV DC converter has a linear behavior of the form $V_{out}=mV_{in}+b$, where *m* and *b* vary according to the connected load. The characterized values of *m* and *b* are shown in Figure 3. For example, in the case of shock: If the DC-HV DC converter has an input of 2.7 V and it has a connected load of 80%, which we interpreted as having 64 transducers excited to the maximum PRF, then the DC-HV DC converter generates 100 V. However, if the PIG instantly brakes, then with the same 2.7-V input, the DC-HV DC converter would generate almost 130 V at the output, which could damage the chips in the pulse generator circuit. In the case of the hammer effect, for example: If the DC-HV DC converter has 2 V at the input and it has a load of 10%, which we interpreted as having 64 transducers connected to a low PRF, then the DC-HV DC converter generates 90 V. However, if the PIG is accelerated instantaneously, then with the same 2-V input, the DC-HV DC converter generates almost 70 V at the output, so the ultrasound signals cannot be received clearly.

Different ultrasound systems [2,27,28,29,30] use HV power supplies to excite the piezoelectric transducers, but these investigations did not focus on the generation of HV because the application they perform does not require a dynamically variable PRF. Some authors [26,31,32,33] have presented the method of producing the HV power supply for their ultrasound system. However, these customized HV power supplies are too big to be used in a PIG. The DC-HV DC converter device is already used in ultrasound systems to reduce size [23,34]. However, due to the type of application only having portable equipment for manual inspection, no analysis was performed on the effect of HV drop on PRF. Improvements of the HV pulser circuit were focused on the addition of passive elements [8,35], the linearization of the MOSFETs’ response utilizing a DC bias-controlled power MOSFET shunt with parallel inductors and capacitors [36] and the activated protection of the ultrasound receiver of the HV spikes [37]. Due to the type of application using a pulse generator circuit, the improvements mentioned above did not require improving the quality of the HV power supply, since they did not have the problem of dynamically variable PRF.

We chose the proportional-integral-derivative (PID) control scheme due to the linearity of the output of the DC-HV DC converter device. The main problem was determining the tuning process and the control law that would produce better results, mitigating the variations in voltage caused by hammer and shock effects. The tuning process should consider the following variables: the number of transducers, variable PRF, average desirable voltage, voltage range and the maximum voltage. Several in-line PID tuning techniques have been developed [38,39], which adjust the control gains according to the dynamic behavior of the system. However, a technique of this type would consume much computational resource owing to the number of operations required. Furthermore, the time between each control action is minimal in a PIG, because of the velocity of pipeline inspection. In this work, we tuned the PID control off-line considering the variables mentioned above, finding the control law and the gains that give a good performance of the ultrasonic pulse generator for automatic pipeline inspection with a dynamically variable PRF. The three discrete control schemes compared were: (1) PID tuned by simultaneous optimization of several responses (SOSR), (2) PID tuned by a neural network (NN) and (3) PI tuned by the analytical design method (ADM). We selected the SOSR technique [40,41] for the comparison because the experimental designs have proved useful for tuning PID systems where the variables that influence the desired output are known [38]. We chose PID tuned by NN with the control law proposed by [39], since it proved to have excellent results for fixed gains of the PID control, which is what we want to achieve at the end of this work. We selected PI control tuned by ADM for the comparison because it allowed us to discard the limitations of electronic components; it also enables us to force the error sequence to become zero when the closed-loop system is subjected to a specific type of time-domain input after a finite number of sampling periods [42].

This paper presents an improvement of the ultrasonic pulse generator via the PID control, which has global gains for the connected load. The load is interpreted as the variable PRF or the amount of connected piezoelectric transducers. The HV-control proposed improves the thickness estimation and protects the circuits used for HV excitation when the PIG changes its velocity dramatically due to the hammer and shock effects. The main advantage of this control is the use of global gains that allow the correct operation of the PIG for automatic pipeline inspection for any value of PRF between 0 and 600 Hz. Section 2 shows the design of the pulse generator circuit. Section 3 presents the materials and methods used. Section 4 describes the experimental methods and the results obtained. Section 5 presents the discussion generated by this project, and Section 6 outlines the conclusions obtained.

## 2. Ultrasonic Pulse Generator Design

**Figure 4:**
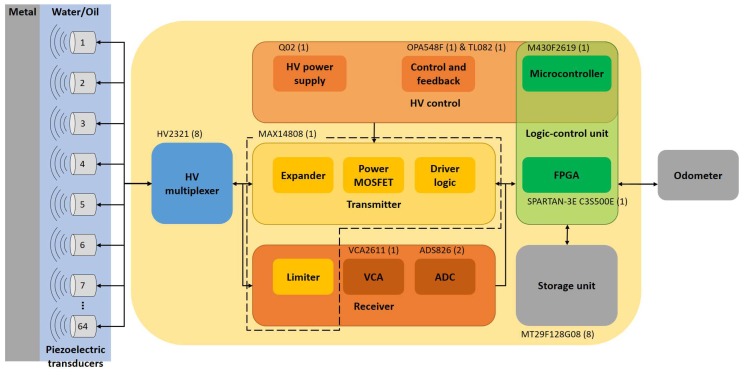
Ultrasound system with the pulse generator control-loop proposal for automatic pipeline inspection.

Figure 4 shows our ultrasound system, proposed for automatic pipeline inspection. The HV multiplexer is the integrated circuit (IC) HV2321. The pulser device is the IC MAX14808, which internally is composed of the limiter an expander, power MOSFETs and the driver logic. The logic-control unit is shared by the FPGA Spartan XC3S500E and the microcontroller M430F2619. The storage system is via the NAND Flash memory chips MT29F128G08 in a RAID-6 architecture [43]. Besides, Figure 4 shows the HV control block proposed; which is composed of the operational amplifiers TL082 and OPA548F. This HV control block improves the thickness estimation and protects the IC MAX14808 when the PRF changes drastically. Although the microcontroller is responsible for computing the control operations, it was already part of the ultrasound system, so it was not considered an added device for the control block of the DC-HV DC converter device.

**Figure 5:**
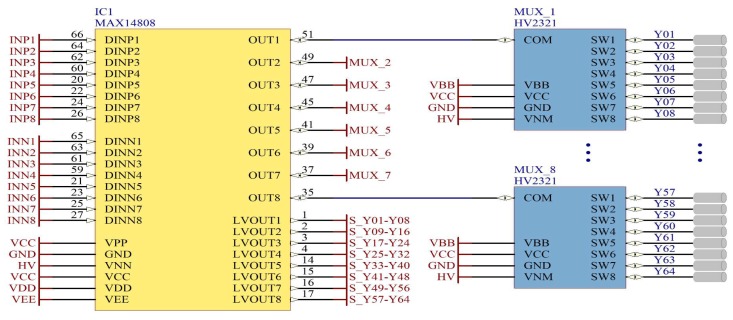
HV pulser device and multiplexer for 64 piezoelectric transducers.

Figure 5 shows the ultrasonic pulse generator circuit, consisting of the pulser device MAX14808, The MAX14808 has embedded over-voltage-protection diodes and an integrated active return-to-zero clamp. Internally, the MAX14808 also has the T/R switch circuit to reject the HV and to obtain the low input voltage of the transducers for the LVOUT pins. An additional advantage of the device MAX14808 is that it does not need to use control MOSFETs to excite the system since it also has them integrated. Each channel of the MAX14808 is multiplexed by eight, via the multiplexer circuit HV2321, allowing us to work with up to 64 piezoelectric transducers using this system. We duplicate the entire system if more transducers were required for the pipeline inspection. The HV2321 is an analog switch with bleed resistors, compatible with applications requiring a large negative voltage. The bleed resistor eliminates voltage built up on capacitive loads of the piezoelectric transducers.

**Figure 6:**
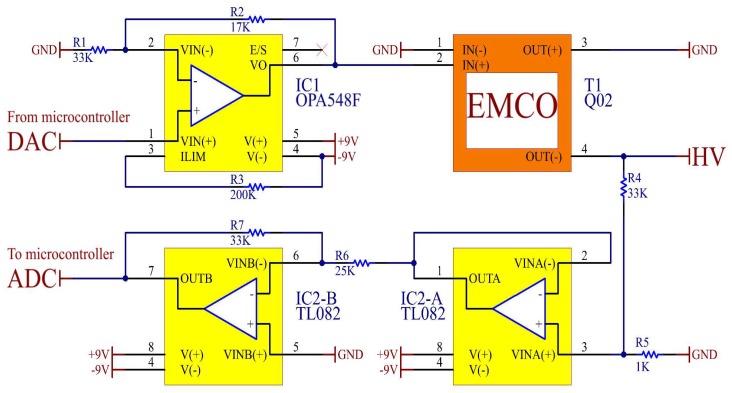
Circuit proposed for controlling the DC-HV DC converter.

The DC-HV DC converter is the EMCO Q02 device; it supplies the negative HV (VNN) for the 64 transducers through the pulser circuit MAX14808. Figure 6 shows the circuit proposed for controlling the HV DC converter. The digital to analog converter (DAC) output of the Texas Instruments microcontroller MSP430F2619 generates the output signal, which ranges from 0–3.3 V with a resolution of 10 bits. The operational amplifier OPA548F amplifies the analog signal of the DAC output, and its main advantage is that it can supply up to 3 A continuously. It is important to mention that we considered the analog control action type, since the PWM control action type could generate contrary effects on the HV pulse. The control feedback is performed using a voltage divider with precision resistors. Subsequently, a voltage follower and a voltage inverter are connected to read the feedback signal with the analog to digital converter (ADC) input of the microcontroller.

## 3. Materials and Methods

We used the development board MAX14808EVKit [44] for the testing. This board can work with different waveforms and adjustable output frequency. Additionally, the MAX14808EVKit has one RC circuit per channel (R = 1 Kohm, and C = 220 pF), the behavior model of which is similar to that of the piezoelectric transducer. The development board also has available connections for HV from an external source, and it is compatible with the development board MAXINT1 [44], which has a SPARTAN-3E FPGA Model XC3S700AN. The FPGA generates the control pulses, and the DC-HV DC converter device EMCO Q02 supplies the HV. The processing unit is the microcontroller M430F2619, manufactured by Texas Instruments. The mixed signal oscilloscope used was the KEYSIGHT Model MSO-X 4154A. The transducers used were the 5-MHz piezoelectric-type.

### 3.1. Characterization of the Transfer Function of the DC-HV DC Converter

**Figure 7:**
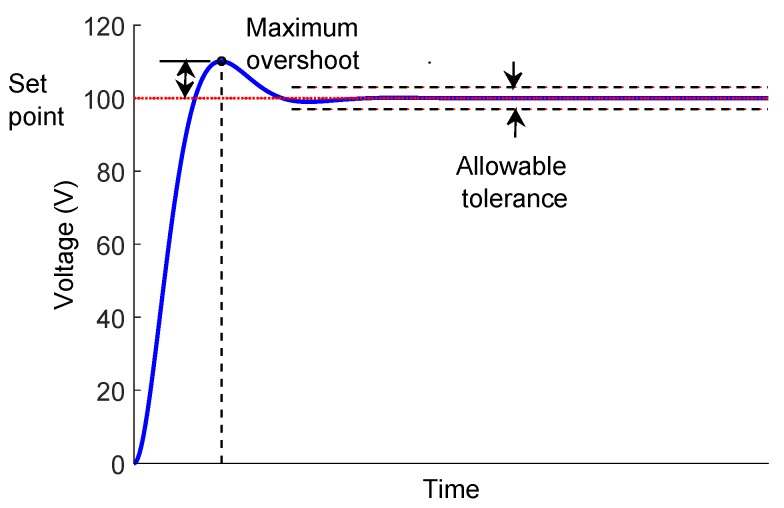
Three primordial characteristics response for the DC-HV DC converter in automatic pipeline inspection.

For the ultrasonic pulse generator in automatic pipeline inspection, the HV signal has three primordial characteristics: (1) the maximum voltage (maximum overshoot), (2) the average desirable voltage (set point) and (3) the voltage range (allowable tolerance). Figure 7 shows graphically the characteristics mentioned. The first step for the control tuning was the characterization of the DC-HV DC converter device. We performed the test with a 5-V step input and percentages of 10%, 45% and 80% of a connected static load.

For the characterization, we considered an auto regressive exogenous (ARX) model for the simulation of the DC-HV DC device dynamics. Equation (1) shows the ARX model, where V(z) is the discrete measured output voltage of the DC-HV DC converter device, $B(z^{-1})$ is the coefficients vector that considers the effect of voltage input at past moments, vector $A(z^{-1})$ considers the effect of previous DC-HV DC converter outputs on the current, U(z) is the vector of the input voltage and E(z) is the additive noise that appears in the system. The advantage of the ARX model is that it takes into account the input and the additive noise influenced by the A(z−1), which is the autoregressive operator of the DC-HV DC device [45].

(1) $$V\left(z\right)=\frac{B(z^{-1})}{1-A(z^{-1})}U\left(z\right)+\frac{1}{1-A(z^{-1})}E\left(z\right)$$

The transfer function was estimated using the least squares technique. We expressed the ARX model as a function of the parameters and measurements. Equation (2) shows this expression, where *k* is the number of observations, $E^{T}\left(k\right)$ is the vector of the error in the measurements, $P^{T}$ is the parameter vector, $Z^{T}\left(k\right)$ is the matrix of measurements of (2m+1)k dimension and *m* is the order model. The matrix $Z^{T}\left(k\right)$ is shown in Equation (3), where *u* is the input voltage and *y* is the output voltage.

(2) $$V^{T}\left(k\right)=P^{T}Z^{T}\left(k\right)+E^{T}\left(k\right)$$

(3) $$Z^{T}\left(k\right)=\left[\begin{array}{cccc} u(1) & u(2) & \ldots & u(k)\\ y(0) & y(2) & \ldots & y(k-1)\\ u(0) & u(1) & \ldots & u(k-1)\\ \vdots & \vdots & \vdots & \vdots \\ y(1-m) & y(2-m) & \ldots & y(k-m)\\ u(1-m) & u(2-m) & \ldots & u(k-m) \end{array}\right]$$

With the analysis of the mean squared error (MSE) of the ARX model of Equation (2), we obtained the least squares estimate shown in Equation (4). Table 1 shows the transfer functions obtained for the responses of 10%, 45% and 80% of static load connected.

(4) $$\hat{P}\left(k\right)={\left(Z^{T}\left(k\right)Z\left(k\right)\right)}^{-1}Z^{T}\left(k\right)Y\left(k\right)$$

### 3.2. Control Scheme Selection

The second step was to propose three different tuning schemes for the PID control. The three discrete control schemes compared were: (1) PID tuned by simultaneous optimization of several responses (SOSR), (2) PID tuned by a neural network (NN) and (3) PI tuned by analytical the design method (ADM) for pole elimination. For these three discrete control schemes, the following conditions were maintained: (1) The microcontroller M430F2619 generates via an internal DAC module the analog voltage for the operational amplifier OPA548F. (2) The OPA548F supplies the DC-HV DC converter device EMCO Q02. (3) The microcontroller performs an estimation of action control every 224 μs, which is ten-times faster than the converter transient response and the minimal time for the action control computing in the microcontroller. (4) The action control (τ(n)) has an upper value of 1023 digital units and a lower value of 0. (5) If the action control is lower than 0, then the microcontroller generates the value of 0 digital units. (6) If the action control is greater than 1023 digital units, then the microcontroller generates the value of 1023 and is considered saturated.

#### 3.2.1. PID Tuning by Simultaneous Optimization of Several Responses (SOSR)

The first method for tuning was SOSR [40,41]. The advantage of this method is that it considers all factors of the implementation, such as the saturation of the control and the dead zone for the integral action. We proposed a dead zone for the integral action of this tuning method due to problems with overshooting in discrete controllers [46], so we activated the integral action only when the voltage of the DC-HV DC converter device was lower than the absolute value of 5 V to the set point. Equation (5) shows the control law, where Kp is the proportional gain, Kd the derivative gain and Ki the integral gain. *n* appoints the present moment for the computation; therefore, n−1 indicates a result of the previous cycle.

(5) $$\tau \left(n\right)=\left\{\begin{array}{cc} \tau (n-1)+Kp\;e(n)+Kd\;(e(n)-e(n-1))+Ki\;\sum e(n) & \mathrm{if}\;\;|e(n)|\leq 5v\\ \tau (n-1)+Kp\;e(n)+Kd\;(e(n)-e(n-1)),\;\;\sum e(n)=0 & \mathrm{if}\;\;|e(n)|>5v \end{array}\right.$$

For the experiment, we performed a fractional factorial design of 1/2 with four factors. In this way, only half of the possible combinations were tested, decreasing from sixteen to eight. However, there were two repetitions, meaning that we repeated the runs two times; also, we considered four center points in the test, giving a total of twenty runs. The factors were Kp, Kd, Ki and the percentage of the connected load. The Kp gain was between the limiting values of 0.1 and 10.00; the Kd gain was between 0.01 and 3.0; the Ki gain was between 0.0 and 1.0; and the percentage of the connected load was between 10% and 80%. In the center points, we tested the system with the median value of each factor.

Table 2 shows the experimental results. The order of the data corresponds to the order in which we performed the tests. The set point was 100 V. The responses considered for the experimental design were the maximum voltage (Vmax), the average set point (Vmean) in steady state and the voltage range (Vrange) in which the DC-HV DC converter voltage output oscillates with the applied control.

**Figure 8:**
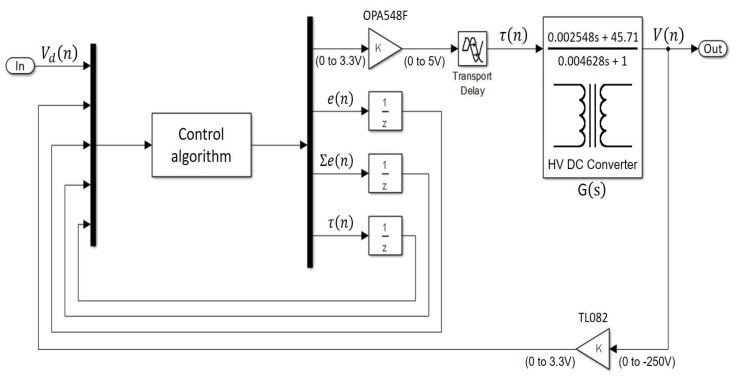
Experimental setup for the tuning process by simultaneous optimization of several responses (SOSR).

In the first run of each factor combination, we carried out the experimental runs without the connection of the pulser device MAX14808, only connecting a static load. In this way, we made sure that the tuning tests would not damage the devices of the ultrasonic system. In the second run of each factor combination, we carried out the runs via simulation tools with Simulink^TM^ and MATLAB^TM^. For the simulation, we used the three discrete ARX models of the DC-HV DC converter device (showed in Table 1), the control algorithm and a time of 224 μs between each step of the simulation. Figure 8 shows the experimental setup.

For the analysis, we used a certainty level of 95%, and we excluded the connected load factor for this analysis. In this way, the analysis only considers the variation of the voltage output as a function of the control gains. Equation (6) shows the approximation for Vmax, which had an $R^{2}$ of 87.66, where $R^{2}$ is the coefficient of determination (adjustment ability), and it provides a measure of the likely accuracy of future outcomes, predicted by the model [38]. Equation (7) shows the approximation for Vmean, with an $R^{2}$ value of 94.14. Equation (8) shows the approximation for Vrange, with an $R^{2}$ value of 93.53. It is important to mention that in this work, the discussion is limited to showing the control tuning method for the DC-HV DC converter device, considering all the technical parameters for its implementation. The range of control gains and the effect of interaction between control parameters are essential points that can be analyzed in the future; however, we did not carry out this analysis as a part of this work.

(6) $$\begin{array}{cc} \hat{Vmax}=149.38 & +6.87\;Kp-13.13\;Kd+0.62\;Ki-0.62\;KpKi\\ & +13.12\;KpKd-5.62\;KiKd-25.62\;KpKdKi \end{array}$$

(7) $$\begin{array}{cc} \hat{Vmean}=101.231 & +1.475\;Kp-2.056\;Kd-1.713\;Ki+1.556\;KpKi\\ & +2.462\;KpKd-2.225\;KdKi+1.769\;KpKdKi \end{array}$$

(8) $$\begin{array}{cc} \hat{Vrange}=8.063 & +1.088\;Kp+0.038\;Kd+3.638\;Ki-2.737\;KpKi\\ & -0.937\;KpKd+0.212\;KdKi-0.362\;KpKdKi \end{array}$$

For the PID control tuning, we used the technique of Derringer and Suich of simultaneous optimization [40,41]. Technicians recommended using an adjusted $R^{2}$ greater than 70% for each model to apply this optimization technique; in addition, each model must fulfill the normality, constant variance and independence of the residuals [47]. Derringer and Suich’s method allows us to define a function in the factor space that computes the composite desirability ($d_{G}$) of the product at each point. The objective of the method is to maximize $d_{G}$ to find the optimal point. The first step is to transform each predicted response *Y* into a value within a function of desirability known as *d*. The transformation for Vmax is shown in Equation (9). Whereas the goal for Vmax was to minimize its value, for $d_{2}$, the goal was to reach a target value of 100 V, with a desirability function between 95 and 105, and for $d_{3}$, the goal was to minimize the voltage range.

(9) $$d_{1}=\left\{\begin{array}{cc} \;1 & \hat{Vmax}<100\\ \;\left(\frac{110-\hat{Vmax}}{110-100}\right) & 100\leq \hat{Vmax}\leq 110\\ \;0 & \hat{Vmax}>110 \end{array}\right.$$

(10) $$d_{2}=\left\{\begin{array}{cc} \;0 & \hat{Vmean}<95\\ \;\left(\frac{\hat{Vmean}-95}{100-95}\right) & 95\leq \hat{Vmean}\leq 100\\ \;\left(\frac{105-\hat{Vmean}}{105-100}\right) & 100\leq \hat{mean}\leq 105\\ \;0 & \hat{Vmean}>105\\ \; \end{array}\right.$$

(11) $$d_{3}=\left\{\begin{array}{cc} \;1 & \hat{Vrange}<1\\ \;\left(\frac{5-\hat{Vrange}}{5-1}\right) & 1\leq \hat{Vrange}\leq 5\\ \;0 & \hat{Vrange}>5 \end{array}\right.$$

Finally, we computed all the possible multiplications of the three desirability functions $d_{1}$, $d_{2}$ and $d_{3}$ to find $d_{G}$ where it presents the maximum value. The results gave values for Kp = 0.1, Kd = 3.0 and Ki = 0.0606.

#### 3.2.2. PID Tuning by Neural Network

**Figure 9:**
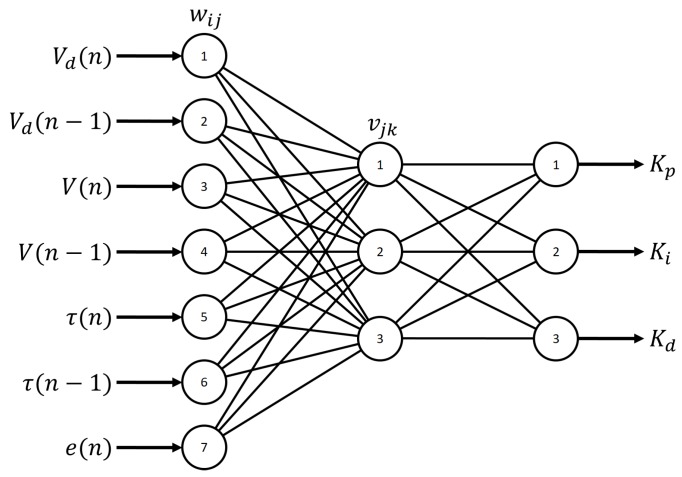
Three-layer artificial neural network topology for tuning the PID control [39].

The PID tuning by NN is a nondeterministic method that is useful for all possible scenarios. The algorithm used for the tuning process employs the back-propagation method. For the tuning process, we used the NN and the PID equation proposed by Hernández et al. [39]. The NN topology is shown in Figure 9, where $V_{d}\left(n\right)$ and $V_{d}\left(n-1\right)$ are reference inputs of the desired voltage, V(n) and V(n−1) are reference outputs of the real voltage outputs, $w_{ij}$ is the weight value of the hidden layer and $v_{jk}$ is the weight value of the output layer.

**Figure 10:**
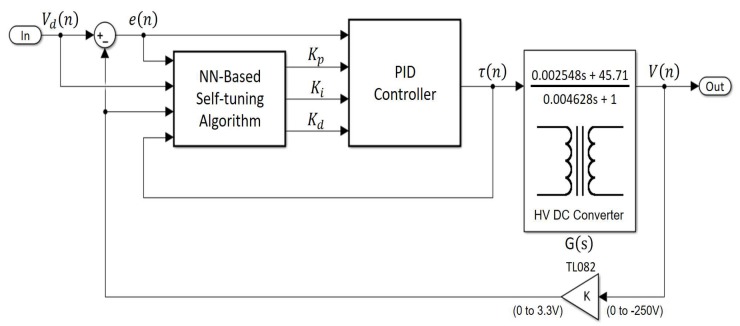
Experimental setup for the tuning process by the neural network (NN).

The back-propagation algorithm looks for the minimum of the error function in weight space using the method of gradient descent. The combination of weights that minimizes the error function is considered to be a solution to the learning problem. The PID control law is shown in Equation (12), where τ(n) and τ(n−1) correspond to the control action signals, e(n) represents the voltage error, Kp is the proportional gain, Ki is the integral gain, Kd is the derivative gain and *n* is the sample time. The experimental setup of the auto-tuning control with NN is shown in Figure 10.

(12) τ(n)=τ(n−1)+Kp(e(n)−e(n−1))+Kd(e(n)−2e(n−1)+e(n−2))+Kie(n)

The main difference in our application versus that of Hernández et al. [39] was that we found a set of gains that allowed us to achieve the best results for the different load conditions without changing in-line the PID control gains. To achieve this, we used 3 epochs for the training of the NN. At each epoch, we changed the transfer function of the DC-HV DC converter (10%, 45% and 80% of static load connected). The global gains obtained were as follows: Kp = 8, Kd = 8 and Ki = 8.

#### 3.2.3. PID Tuning by Analytical Design Method

**Figure 11:**
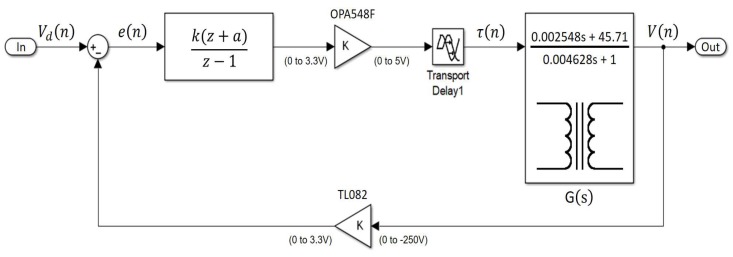
Experimental setup for the tuning process by the analytical design method (ADM).

The third method for tuning the controller was the ADM for a discrete time [42]. Figure 11 shows the experimental setup for the analytical design method. Equation (13) shows the transfer function of the digital controller. This method aims to eliminate the pole of the G(z) (which is z=0.9528) and replace it with the pole z=1, also finding a gain *k* that allows the system to have the desired response. Equations (14) and (15) show how to calculate the zero value *a* that eliminates the pole and how to calculate the gain *k*, respectively. *T* is the sample period of 224 μs between each step of the simulation. Kp and Ki are the proportional gain and integral gain, respectively.

(13) $$G_{P}\left(z\right)=\frac{k(z+a)}{z-1}$$

(14) $$a=\frac{KiT-2Kp}{2Kp+KiT}$$

(15) $$k=\frac{KiT+2Kp}{2}$$

We selected for the ADM the PI control because it has the advantage of eliminating the error in steady state in response to step type inputs. The transfer function of the digital controller is PI, and it is shown in Equation (16).

(16) $$G_{D}\left(z\right)=Kp+Ki\;\frac{0.5T\;(z+1)}{z-1}$$

The first step for this tuning process was to find Ki. Because the DC-HV DC converter transfer function is of order one, its error in steady state against a step input is $e_{ss}=\frac{1}{Kv}$. We considered the additional constraint of the static velocity error constant kv with the value of 900; in this way, the error cannot be higher than de0.11 V for a step input of 100 V. We applied the final value theorem to the function $G_{D}\left(z\right)G\left(z\right)$ to find Kv. The error in steady state is expressed in Equation (17). Ki gave a value of 19.6893.

(17) $$Kv=\lim_{z\to 1}\left[\frac{1-z^{-1}}{T}G_{D}\left(z\right)G\left(z\right)\right]=45.71Ki$$

The second step was to find Kp through Equation (14), equal to −0.9528, as shown in the Equation (18).

(18) $$\frac{KiT-2Kp}{2Kp+KiT}=-0.9528$$

Solving for this system, Kp gives a value of 0.0911. Once we found Kp and Ki, they were substituted into Equations (14) and (15), giving the values of *a* and *k* as −0.9528 and 0.0933, respectively.

## 4. Results

**Figure 12:**
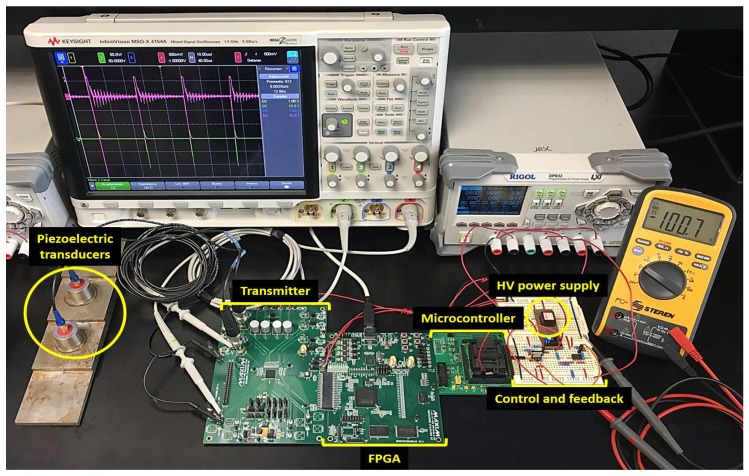
Ultrasound system used for experimental testing.

We grouped our tests into three stages. The first stage was the selection of the control scheme; this stage was carried out by simulation. In the second stage, we tested the behavior of the control scheme for different step inputs. In the third stage, we tested the behavior of the system for different frequencies and amplitudes. The second and third stages were carried out using the ultrasonic system shown in Figure 12. We tested the eight channels of the pulser circuit, connecting two channels with 5-MHz piezoelectric transducers of the straight beam for immersion and the other six channels with the RC circuit included in the development board MAX14808EVSYS.

### 4.1. Selecting the Control Scheme

**Figure 13:**
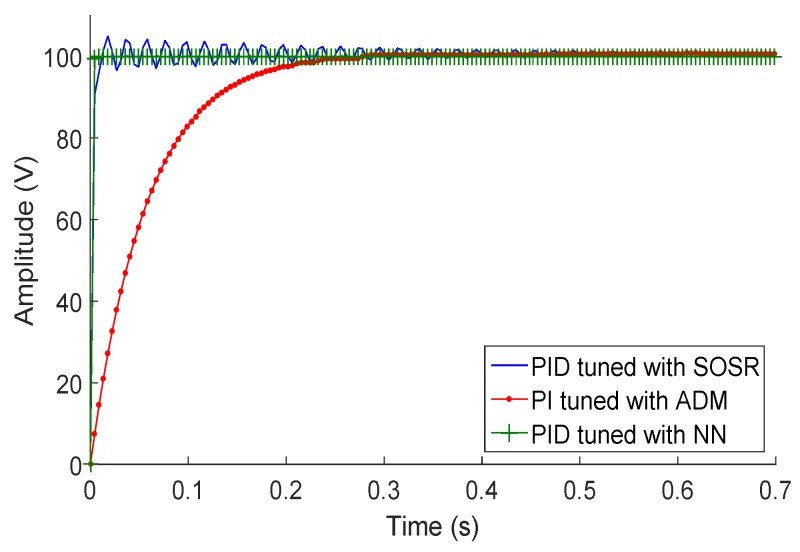
Step response for an input of 100 V.

We performed two tests for the selection of the control scheme. The first test was for a 10% static connected load and 100-V step input of each control scheme. We chose the transfer function of 10% connected load because it was the function that had the most variation of the output voltage in relation to the input. The second test consisted of analyzing the behavior of the three control schemes when the PIG suffers from the hammer effect and braking. Figure 13 shows the behavior of the three control schemes for a 100-V step input and the transfer function of 10% connected load. Observing that the three control schemes have different behaviors, the PI control is the scheme that best fits the set point; however, it is a control with a slow response compared to the other two schemes. The PID tuned by SOSR is fast and fits the set point, but it has an over-voltage, which is within the established range of Equation (11). The PID tuned by NN is fast and adjusts to a fixed value; however, it presents an offset in relation with the set point.

**Figure 14:**
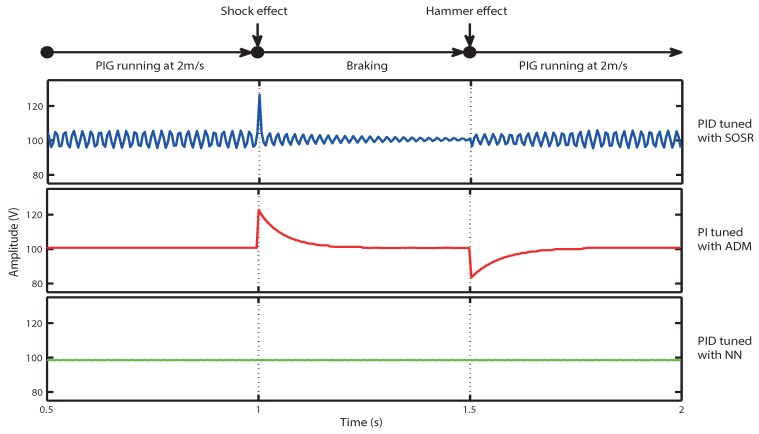
Shock and hammer effect responses for the control schemes.

The second test for the selection of the control scheme was the analysis of the control scheme responses for the shock and hammer effects. The most critical velocity change for the ultrasound system is the instantaneous braking of the PIG, which occurs when the PIG crashes due to a dent or deformation of the pipeline. According to our observations, the DC-HV DC converter (see Figure 3) raises its output voltage immediately, damaging the circuits of the ultrasonic pulser. The hammer effect causes a voltage drop that affects the amplitude of the signals received. This means that the information of a section of the pipeline may be lost until the HV approaches its pre-adjusted value. Figure 14 illustrates the testing of the three control schemes for a shock effect at 1 s and a hammer effect at 1.5 s. The simulation step was every 5 ms, and the resolution in the *y* axis was 0.0001 V.

For the shock effect at time *t* of 1 s, the simulation showed the behavior in the moment when the PIG went from being a speed of 2 m/s to practically stationary. The most critical parameter, in this case, is the excess energy when the output voltage is over 105 V (until the voltage is adjusted back to 100 V). In this case, a lower energy is best, since the control scheme should protect the chips of the pulser circuit from an over-voltage. Equation (19) shows how we computed the excess energy [11].

(19) $$E=\sum_{n=a}^{b}{\left(x\left(n\right)-100\right)}^{2}$$

In Equation (19), *a* is the initial moment from which the summation begins, and *b* is the final moment for data acquisition. For the shock effect, we changed the transfer function from 80% to 10% of the connected load at 1 s of the simulation. Therefore, for Equation (19), a= 200, and data capture was stopped at 1.5 s, reaching b= 300. For the hammer effect, we changed the transfer function from 10% to 80% of the connected load at 1.5 s of the simulation. For the DC-HV DC converter, this is a drastic change of the load that produces a voltage drop; in this case, the control scheme that has the shortest recovery time (the time to return the voltage to 100 V with a limit of ±5 V) is considered the best. Data capture was stopped when the voltage reached the lower value of 95 V.

Table 3 shows the comparison of the three control schemes. In the case of the excess energy generated by the shock effect, the PI control by ADM was the one that generated the most energy, for which reason it was dismissed as a possible control scheme. In the case of the maximum overshoot for the shock effect, the PID control tuned by SOSR generated the highest voltage value, reaching a value of 127.71 V, with an excess of 27.71 V that could damage the chips of the pulse generator circuit. In the case of the recovery time, the control scheme that suffered a voltage drop below 95 V was the PI tuned by ADM; the other two control schemes supported the load change without exceeding the limits for the hammer effect. Based on the results of the two tests, we selected the PID control tuned by NN for our ultrasound system. This scheme showed no excess energy in the response of the shock effect, did not generate any overshoot to the shock effect and did not generate a voltage drop in response to the hammer effect of the PIG. These results show that the PID control tuned by the NN control scheme assures the protection of the chips that are part of the pulse generator circuit against over-voltage for hammer and shock effects.

### 4.2. Control Testing of Step Input

**Figure 15:**
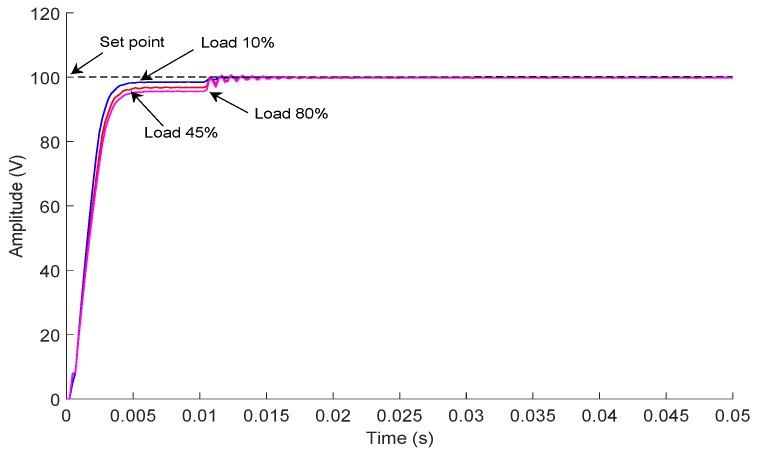
Response of the control to a 100-V set point with different connected load values.

Once the control scheme was selected, the second test stage consisted of recording the behavior of the PID control tuned by NN, fixing a set point of 100 V and varying the connected load to the output of the DC-HV DC converter device. Figure 15 shows the results obtained for the loading percentages of 10%, 45% and 80%, and no overshoot was observed.

Figure 15 also shows that there was an offset between the set point and the voltage obtained. We eliminated this offset through a self-adjustment of the set point at 10 ms. Although in the work [39], the authors presented this control scheme as a PID, we detected that the control law did not have an integral action, which is what generated the previously-described offset; however, the tuning method and the structure of the control presented the best results for our application. The second test stage showed the capacity of the PID tuned by NN for controlling and monitoring the delivered power quantity in spite of the variation of the connected load.

### 4.3. Control Testing for Different Frequencies and Amplitudes

**Figure 16:**
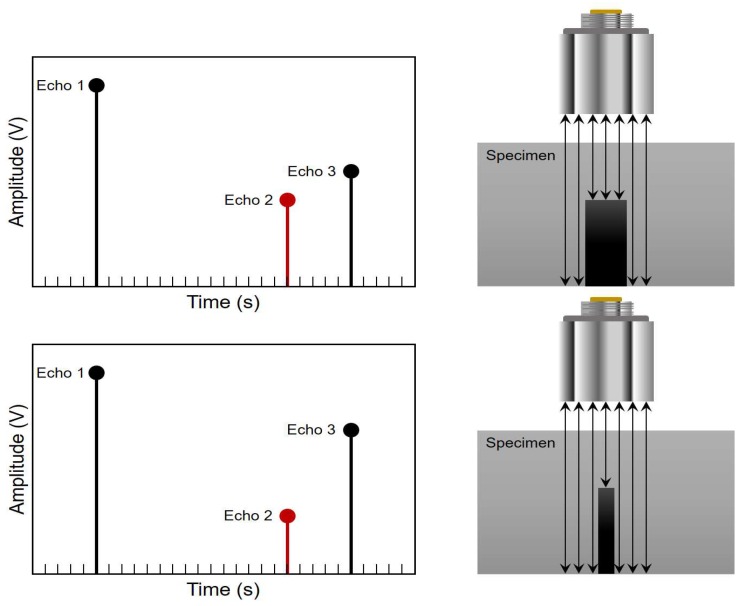
Reduction of the echo amplitude of the posterior wall of the pipe in relation to the size of a defect.

For the automatic pipeline inspection by the pulse-echo method in immersion, the estimation of the size of defects that are smaller than the size of the ultrasound beam depends on the amount of energy that is reflected in the defect (see Figure 16, Echo 2). If the defect is small, an echo with a low amplitude appears between the internal and external wall echoes (Figure 16, Echoes 1 and 3); but if the defect is significant, an echo appears with a higher amplitude. If there is no control of the amount of energy emitted in each ultrasound echo, then when interpreting the post-processing data, it will be difficult to determine the size of the defect found.

**Figure 17:**
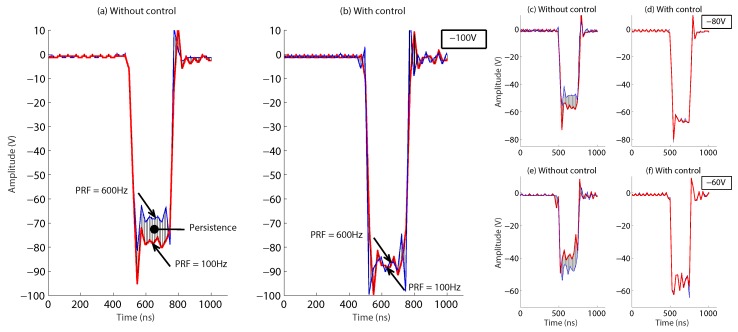
Comparison of the quality of the HV pulse with the proposed control and without the control, for different values of voltage and different frequencies. Set point at −100 V, (**a**) without control, (**b**) with control. Set point at −80 V, (**c**) without control, (**d**) with control. Set point at −60 V, (**e**) without control, (**f**) with control.

Figure 17 shows the third test stage performed with the ultrasound system considering all the conditions for the inspection of the pipeline, which are: 64 transducers with a maximum PRF of 600 Hz and an excitation voltage between −50 V and −100 V. For this test, with the intention of not affecting the response signal obtained and to show the improvement of the ultrasonic pulse generator, we did not consider the backup capacitors for HV, nor rectification diodes. The PRF of 600 Hz shown in Figure 17 considers the excitation of eight transducers at the same time. Therefore, the rate at which the pulser circuit worked to excite the 64 piezoelectric transducers was up to 4.8 kHz, which is eight-times faster due to multiplexing devices HV2321. Figure 17 also shows how with the PID control scheme, the excitation pulse of the transducers reaches the required amplitude value. For example, with the set point of −100 V and without the control (see Figure 17a), the output voltage of the DC-HV DC converter dropped dramatically when the PRF increased (the red line shows the response for a PRF of 100 Hz). The voltage drop approached 20 V for a PRF of 600 Hz (the blue line shows the response for a PRF of 600 Hz). However, with the PID control (see Figure 17b), the output of the DC-HV DC converter was able to supply the −100 V in an instant.

Figure 17 also shows the persistence of the HV pulses obtained for the different PRFs. With the control, the HV pulses maintained practically the same amplitude and shape; however, without the control, the amplitude of the HV pulses reached a maximum at a PRF value of 100 Hz, but as the frequency of the PRF was increased to 600 Hz, the amplitude of the HV pulse dropped. This effect was similar for tests from −50 V–−100 V. Figure 17e,f shows the waveforms for −60 V and Figure 17c,d for −80 V. The HV pulse can now be improved by high-frequency passive elements that work at 200-ns intervals of the pulse length to reduce the drop in amplitude. The third stage test showed the capacity of the PID tuned by NN for amplifying the HV to the set point desired and reducing the problems with failures in wall thickness estimation.

## 5. Discussion

The linear behavior of the DC-HV DC converter makes the use of a PID controller possible, but the problem that we resolved was determining the tuning process and the control law that would allow us to obtain better results and mitigate the variations of voltage caused by hammer and shock effects. With the improvement implemented in the DC-HV DC converter device, the ultrasonic pulse generator is capable of operating at a variable PRF, within the range of 0–600 Hz and generating pulses at a voltage of −20 V–−100 V.

The inspection of pipelines for oil and water distribution is a task with a high degree of interest in the industrial sector. The development of more compact subsystems to reduce the weight of the PIG is necessary. The researchers are focused on developing more flexible technology for different diameters that is as economical and compact as possible. In this article, we present an alternative method to safely replace the HV power supply used in the ultrasound systems for automatic pipeline inspection. This improvement does not reduce the problems presented by the automatic inspection such as the chattering, nor does it improve the capacities of the straight beam; however, it gives the certainty that the voltage used for the excitation of the transducers is controlled, and it will not damage the chips of the pulse generator.

We grouped our tests into three stages. In the first stage, we proved that the PID control tuned by NN assures the protection of the chips that are part of the pulse generator circuit against over-voltage for hammer and shock effects. In the second stage, we proved that the control scheme is effective in controlling the delivered power quantity in spite of the variation of the connected load, which we interpreted as the dynamically-variable PRF. In the third stage, we proved that the control scheme amplifies the HV to the set point desired and reduces the problems with failures in wall thickness estimation. In this way, the ultrasound system is useful for inspecting pipelines employing the PIG. The control applied to the converter allows a reduction in the size of the ultrasound systems by using only an amplifier encapsulation, which is controlled in order to maintain the parameters of Vmax, Vmean, and Vrange at the desired values.

**Figure 18:**
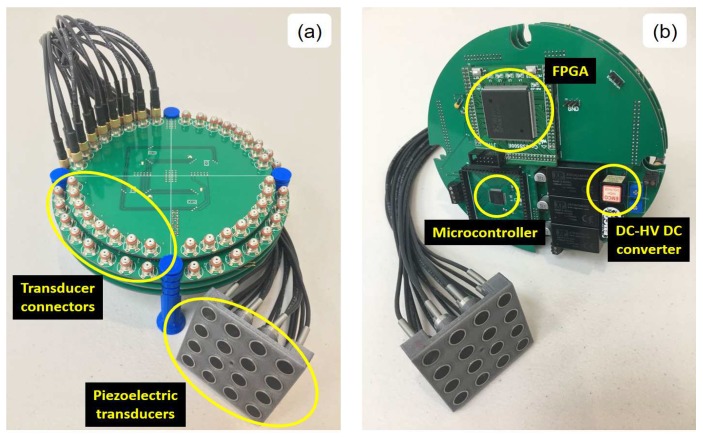
Ultrasound system designed for automatic pipeline inspection. (**a**) Top view and (**b**) bottom view.

Figure 18 shows the ultrasound system designed for automatic pipeline inspection; the printed circuit boards have a circular shape with a diameter of 150 mm in length due to the cylindrical body shape of the PIG (see Figure 1). Figure 18a shows in the top part the arrangement of the 64 connectors where the 5-MHz immersion transducers are connected. Figure 18b shows the bottom part of the system, where the FPGA, the microcontroller and DC-HV DC converter are.

This improvement to the pulse generator circuit gives versatility not only to portable devices, but also to larger devices; since the variation of the PRF can occur in inspection stations, where the technician proposes trajectories for inspection according to the shape of the specimen. Furthermore, as mentioned earlier in the Introduction, this device can also be used for light sources, printers and capacitor charging, where applications can present a constant load variation and control of the DC-HV DC converter is necessary. The next step for our research is to develop new and more compact ultrasound systems, ensuring that the excitation voltage can be controlled efficiently without generating voltages that could damage the prototype chips, therefore reducing the costs of development.

## 6. Conclusions

The construction of more robust technology for the sub-systems of the PIG is an area of very recent interest [21], such as the case of the ultrasound systems for inspection, where they not only must ensure measurements are taken correctly, but must also consider the effects that may occur when the pipeline inspection is carried out. In this sense, we present the improvement of an ultrasonic pulse generator that makes it useful for automatic pipeline inspection. To achieve this, we first defined the crucial parameters of the high voltage of a DC-HV DC converter, which was later characterized by the least squares method, and we compared the three control schemes.

To compare the three control schemes, we considered different scenarios in which the HV-DC HV converter was affected by the shock and hammer effects; in this way, we were discarding the control schemes until the final selection of the PID control tuned by NN. Finally, we realized a series of tests for the adjustment of the control scheme, because the original scheme did not consider an effective integral action. The main advantages that our improvement to the ultrasound system presents are: (1) controllable amplification of ultrasound signals during the automatic inspection, (2) protection of the chips that are part of the pulse generator circuit against over-voltage, (3) reduction of the error in the estimation of thicknesses and defects and (4) control and monitoring of the delivered power quantity.

## Figures and Tables

**Table 1:** Transfer functions of the DC-HV DC converter for a connected load of 10%, 45% and 80%.

% Connected Load	Continuous	Discrete
10	$\frac{0.002548s\;+45.71}{0.004628s+1}*e^{-0.000328s}$	$\frac{0.5505z^{-1}-0.4716z^{-2}}{1-0.9983z^{-1}}*z^{-41}$
45	$\frac{0.0036s\;+40.58}{0.004211s+1}*e^{-0.000392s}$	$\frac{0.8568z^{-1}-0.7797z^{-2}}{1-0.9981z^{-1}}*z^{-49}$
80	$\frac{0.003438s\;+37.5}{0.004013s+1}*e^{-0.000304s}$	$\frac{0.8568z^{-1}-0.7821z^{-2}}{1-0.9980z^{-1}}*z^{-38}$

**Table 2:** Fractional factorial design with the factors Kp, Kd, Ki and the percentage of connected load (%). The responses considered were the maximum voltage (Vmax), the average set point (Vmean), and the voltage range (Vrange).

Kp	Ki	Kd	% Load	Vmax	Vmean	Vrange
10.00	0.0	0.010	80	125.0	102.00	9.0
5.05	0.5	1.505	45	162.5	102.50	6.0
5.05	0.5	1.505	45	162.5	102.50	6.0
10.00	1.0	0.010	10	200.0	101.70	11.7
0.10	1.0	0.010	80	150.0	105.00	11.8
10.00	1.0	3.000	80	125.0	102.50	9.0
10.00	0.0	3.000	10	187.5	103.95	7.5
0.10	1.0	3.000	10	137.5	87.95	15.0
0.10	0.0	3.000	80	95.0	102.50	1.0
0.10	0.0	0.010	10	187.5	103.60	0.2
10.00	1.0	3.000	80	125.0	102.50	9.0
10.00	0.0	0.010	80	125.0	102.00	9.0
0.10	1.0	3.000	10	137.5	88.00	14.8
5.05	0.5	1.505	45	162.5	103.50	6.0
0.10	0.0	0.010	10	187.5	103.50	0.2
10.00	1.0	0.010	10	175.0	103.50	10.5
5.05	0.5	1.505	45	162.5	103.50	6.0
10.00	0.0	3.000	10	187.5	103.50	7.5
0.10	1.0	0.010	80	150.0	105.00	11.8
0.10	0.0	3.000	80	95.0	102.50	1.0

**Table 3:** Metrics for the control scheme comparison for hammer and shock effects.

Control Scheme	Shock Effect		Hammer Effect
	Energy (*E*) ± 0.01 (*E*)	Maximum Overshoot (V) ± 0.0001 (V)	Recovery Time (ms) ± 5 (ms)
PID tuned with SOSR	467.45	127.7106	0
PI tuned with ADM	6335.32	122.7571	90
PID tuned with NN	0	98.5457	0

## References

[1] Sharma K., Singh S., Dubey P.K. (2017). Design of Low Cost Broadband Ultrasonic Pulser–Receiver. MAPAN.

[2] Qiu W., Yu Y., Tsang F.K., Sun L. (2012). A Multifunctional, Reconfigurable Pulse Generator for High-Frequency Ultrasound Imaging. IEEE Trans. Ultrason. Ferroelectr. Freq. Control.

[3] Svilainis L., Chaziachmetovas A., Dumbrava V. (2015). Half bridge topology 500 V pulser for ultrasonic transducer excitation. Ultrasonics.

[4] Haider B. Power Drive Circuits for Diagnostic Medical Ultrasound. Proceedings of the 18th International Symposium on Power Semiconductor Devices and IC’s.

[5] Lei H., Huang Z., Liang W., Mao Y., Que P.W. (2009). Ultrasonic Pig for Submarine Oil Pipeline Corrosion Inspection. Russ. J. Nondestruct. Test..

[6] C. de Normalizacion de Petroleos Mexicanos e Instituciones Subsidiarias (2012). Inspeccion de Ductos de Transporte Mediante Equipos Instrumentados.

[7] The American Society of Mechanical Engineers—ASME (2006). B31.4 Pipelines Transportation Systems for Liquid Hydrocarbons and Other Liquids.

[8] Hayward G. (1985). The influence of pulser parameters on the transmission response of piezoelectric transducers. Ultrasonics.

[9] Cardoso G., Saniie J. (2005). Ultrasonic Data Compression via Parameter Estimation. IEEE Trans. Ultrason. Ferroelectr. Freq. Control.

[10] Alobaidi W.M., Kintner C.E., Alkuam E.A., Sasaki K., Yusa N., Hashizume H., Sandgren E. (2018). Experimental Evaluation of Novel Hybrid Microwave/Ultrasonic Technique to Locate and Characterize Pipe Wall Thinning. J. Press. Vessel Technol..

[11] Guofeng D., Kong Q., Zhou H., Gu H. (2017). Multiple cracks detection in pipeline using damage index matrix based on piezoceramic transducer-enabled stress wave propagation. Sensors.

[12] Canavese G., Scaltrito L., Ferrero S., Pirri C.F., Cocuzza M., Pirola M., Corbellini S., Ghione G., Ramella C., Verga F. (2015). A novel smart caliper foam pig for low-cost pipeline inspection—Part A: Design and laboratory characterization. J. Pet. Sci. Eng..

[13] Alobaidi W.M., Alkuam E.A., Al-Rizzo H.M., Sandgren E. (2015). Applications of Ultrasonic Techniques in Oil and Gas Pipeline Industries: A Review. Am. J. Oper. Res..

[14] Carvalho A.A., Rebello J.M.A., Souza M.P.V., Sagrilo L.V.S., Soares S.D. (2008). Reliability of non-destructive test techniques in the inspection of pipelines used in the oil industry. Int. J. Press. Vessels Pip..

[15] Feng Q., Li R., Nie B., Liu S., Zhao L., Zhang H. (2016). Literature Review: Theory and Application of In-Line Inspection Technologies for Oil and Gas Pipeline Girth Weld Defection. Sensors.

[16] Dobmann G., Barbian O.A., Willems H. (2007). State of the Art of In-Line Nondestructive Weld Inspection of Pipelines by Ultrasonics. Russian J. Nondestruct. Test..

[17] Zhang H., Zhang S., Liu S., Wang Y. (2017). Collisional vibration of PIGs (pipeline inspection gauges) passing through girth welds in pipelines. J. Nat. Gas Sci. Eng..

[18] Zhang H., Zhang S., Liu S., Wang Y., Lin L. (2015). Measurement and analysis of friction and dynamic characteristics of PIG’s sealing disc passing through girth weld in oil and gas pipeline. Measurement.

[19] Zhang H., Zhang S., Liu S., Zhu X., Tang B. (2015). Chatter vibration phenomenon of pipeline inspection gauges (PIGs) in natural gas pipeline. J. Nat. Gas Sci. Eng..

[20] Mazraeh A., Alnaimi F. (2015). Multi-Diameter Pipeline Inspection Gauge for Lang Distance Industrial Application. Int. J. Sci. Eng. Res..

[21] Liu Z., Kleiner Y. (2013). State of the art review of inspection technologies for condition assessment of water pipes. Measurement.

[22] Qiu W., Wang C., Li Y., Zhou J., Yang G., Xiao Y., Feng G., Jin Q., Mu P., Qian M. (2015). A scanning-mode 2D shear wave imaging (s2D-SWI) system for ultrasound elastography. Ultrasonics.

[23] Nava-Balanzar L., Soto-Cajiga J.A., Pedraza-Ortega J.C., Ramos-Arreguin J.M. Development of an Ultrasonic Thickness Measurement Equipment Prototype. Proceedings of the 20th CONIELECOMP.

[24] Soto-Cajiga J.A., Pedraza-Ortega J.C., Rubio-Gonzalez C., Bandala-Sanchez M., Romero-Troncoso R.J. (2012). FPGA-based architecture for real-time data reduction of ultrasound signals. Ultrasonics.

[25] XP Power (2016). Q Series, Isolated, Proportional DC to HV DC Converters.

[26] El-Desouki M.M., Hynynen K. (2011). Driving Circuitry for Focused Ultrasound Noninvasive Surgery and Drug Delivery Applications. Sensors.

[27] Svilainis L., Chaziachmetovas A., Dumbrava V. (2013). Efficient high voltage pulser for piezoelectric air coupled transducer. Ultrasonics.

[28] Brown J.A., Lockwood G.R. (2002). A Low-Cost, High-Performance Pulse Generator for Ultrasound Imaging. IEEE Trans. Ultrason. Ferroelectr. Freq. Control.

[29] Xu X., Yen J.T., Shung K.K. (2007). A Low-Cost Bipolar Pulse Generator for High-Frequency Ultrasound Applications. IEEE Trans. Ultrason. Ferroelectr. Freq. Control.

[30] Canales R.V., Takarabe E.W., Maruyama N., Furukawa C.M. (2008). Digital Ultrasonic System for Internal Corrosion Assessment on Oil Pipelines. ABCM Symposium Series Mechatronics.

[31] Bharath R., Kumar P., Dusa C., Akkala V., Puli S., Ponduri H., Krishna K.D., Rajalakshmi P., Merchant S.N., Mateen M.A. (2015). FPGA-Based Portable Ultrasound Scanning System with Automatic Kidney Detection. J. Imaging.

[32] Gammell P.M., Harris G.R. (2003). IGBT-based Kilovoltage Pulsers for Ultrasound Measurement Applications. IEEE Trans. Ultrason. Ferroelectr. Freq. Control.

[33] Rodríguez J.A., Vitola J., Sandoval S. (2009). Diseño y Construcción de un Sistema de Ultrasonido para la Detección de Discontinuidades en Soldadura. Revista Colombiana de Física.

[34] Wu J.-X., Du Y.-C., Lin C.-H., Chen P.-J., Chen T. A Novel Bipolar Pulse Generator for High-frequency Ultrasound System. Proceedings of the 2013 IEEE International Ultrasonics Symposium (IUS).

[35] Ramos A., San Emeterio J.L., Sanz P.T. (2000). Dependence of pulser driving responses on electrical and motional characteristics of NDE ultrasonic probes. Ultrasonics.

[36] Choi H., Woo P.C., Yeom J.-Y., Yoon C. (2017). Power MOSFET Linearizer of a High-Voltage Power Amplifier for High-Frequency Pulse-Echo Instrumentation. Sensors.

[37] Choi H., Yang H.-C., Shung K.K. (2014). Bipolar-power-transistor-based limiter for high frequency ultrasound imaging systems. Ultrasonics.

[38] Santhakumar M., Asokan T. (2010). A Self-Tuning Proportional-Integral-Derivative Controller for an Autonomous Underwater Vehicle, Based on Taguchi Method. J. Comput. Sci..

[39] Hernández-Alvarado R., García-Valdovinos L., Salgado-Jiménez T., Gómez-Espinosa A., Fonseca-Navarro F. (2016). Neural Network-Based Self-Tuning PID Control for Underwater Vehicles. Sensors.

[40] Derringer G., Suich R. (1980). Simultaneous Optimization of Several Response Variables. J. Qual. Technol..

[41] Derringer G. (1994). A balancing act-optimizing a products properties. Qual. Prog..

[42] Ogata K. (1995). Discrete-Time Control Systems.

[43] Rodríguez-Olivares N.A., Gómez-Hernández A., Nava-Balanzar L., Jiménez-Hernández H., Soto-Cajiga J.A. (2018). FPGA-Based Data Storage System on NAND Flash Memory in RAID 6 Architecture for In-Line Pipeline Inspection Gauges. IEEE Trans. Comput..

[44] Maxim Integrated (2012). MAX14808 Evaluation System Evaluates: MAX14808.

[45] Yuan S., Wu H., Yin C. (2013). State of Charge Estimation Using the Extended Kalman Filter for Battery Management Systems Based on the ARX Battery Model. Energies.

[46] De Wit C.C., Siciliano B., Bastin G. (2012). Theory of Robot Control.

[47] Pulido H.G., Salazar R.V. (2012). Análisis y diseño de Experimentos.

